# Impact of spousal caregiving on frailty index: longitudinal evidence from China Health and Retirement Longitudinal Study

**DOI:** 10.1093/ageing/afaf148

**Published:** 2025-06-08

**Authors:** Mingming Liu, Shanshan Wang

**Affiliations:** Department of Epidemiology and Public Health, University College London, 1-19 Torrington Pl, London, WC1E 6BT, UK; School of Nursing, The Hong Kong Polytechnic University, Hung Hom, The Hong Kong Polytechnic University, Kowloon, Hong Kong

**Keywords:** frailty, spousal caregiving, Chinese population, older people

## Abstract

**Background and objective:**

Research on the impact of spousal caregiving on caregivers’ frailty remains limited. This study aimed to examine this association between spousal caregiving and frailty, explore how this association varies with care intensity, and investigate potential gender differences.

**Methods:**

This study utilized data from four waves of the China Health and Retirement Longitudinal Study, including 3,987 participants aged 50 and above. Frailty was assessed using a composite mean score based on 41 indicators aligned with Rockwood’s frailty criteria. These indicators included self-reported health, medically diagnosed conditions, medical symptoms, functional activities assessment, activities of daily living and instrumental activities of daily living. Samples were stratified by gender, and a growth curve model with random intercepts was employed to examine the associations between spousal caregiving status, care intensity and frailty trajectories over time.

**Results:**

Among females, compared to non-caregivers, spousal caregiving was significantly associated with the increased frailty index when adjusted by all covariates, and frailty accelerated at a higher rate for caregivers. Providing care at all three intensity levels was associated with higher frailty, although depression attenuated these associations. Additionally, caregiving at lower intensity showed an accelerating rate of frailty progression over time. Among males, only providing higher-care intensity was associated with higher frailty.

**Conclusions:**

This study highlights the importance of care intensity as well as the gendered effects of spousal caregiving on frailty—caregiving exacerbates frailty, particularly among females and among higher-intensity male caregivers. Our findings suggest the need for targeted supportive measures to alleviate psychological stress.

## Key Points

- Female spousal caregivers at all three intensity levels were associated with higher frailty.
- Male caregivers providing higher-intensity care also exhibited a higher frailty index compared to their counterparts.
- The rate of frailty progression accelerates over time among female caregivers providing lower-intensity care.
- Depression plays a key role in the association between caregiving intensity and frailty among female caregivers.
- Our findings suggest the need for targeted supportive measures to alleviate psychological stress.

## Introduction

Informal care has become a widely discussed public topic in addressing the insufficiency of formal care resources in the context of rapid population aging. The World Health Organization (WHO) [1], identified the development of appropriate long-term care systems—across home, community and institutional setting—as a key priority [2]. Informal caregiving is highly prevalent, meets the majority of global care needs, and holds significant economic value [3]. In China, increasing life expectancy and declining fertility rates are reshaping demographic structures, with the population aged 60 and older projected to reach 402 million by 2040 [4]. This demographic shift is expected to further escalate the demand for long-term care.

Unlike Western cultures, in China, individual’s behaviours are often constrained or guided by traditional values and norms, particularly Confucianism, whose core view emphasizes family. Influenced by these values, Chinese people are traditionally expected to assume caregiving roles for their family members [5]. This expectation aligns with the governmental ‘90-7-3’ eldercare policy framework, which advocates that ~90% of older adults should receive care at home—primarily from family members, 7% should be supported by community-based hospitals and health centres, and 3% should receive care in institutional settings [6]. Additionally, marriage relationships are also shaped by these cultural values, with mutual caregiving responsibilities throughout marital life [5], positioning spousal caregiving as a critical source of support in later life. Recent demographic and social shifts have further contributed to the growing prevalence of spousal caregiving in China [7]. Although traditional filial piety emphasizes eldercare by adult children, this practice has been weakened due to structural changes—such as the decline of extended and nuclear families resulting from decreasing fertility rates [8]—and geographical dynamics, including rural-to-urban migration of adult children [9]. As a result, adult children are increasingly providing financial support rather than direct physical assistance [7], contributing to the rise of ‘empty nesters’—particularly in rural areas—who are left to care for themselves, or each other [10].

Existing evidence from China consistently suggests that spousal caregiving was associated with negative caregiver outcomes [11–15], with caregiving intensity playing an important role [13, 15]. Confucianism further reinforces gender disparities within family roles, placing primary caregiving responsibilities predominantly on women within a patriarchal context, where they are expected to exhibit altruism and compassion [16]. Furthermore, under traditional views, the unequal division of family labour is assigned to women by men, rather than actively chosen by women themselves. Because they are often excluded from decision-making, caregiving is usually everlasting [17]. As a result, women caregivers are more likely to experience more adverse health outcomes, including depressive symptoms [11, 18], chronic conditions [19] and cognitive decline [15]. According to the transdisciplinary stress model, prolonged exposure to caregiving-related stress accelerates biological aging [20], subsequently increasing the risk of frailty [21].

Frailty, a common age-related condition, results from the cumulative decline across multiple physiological systems, leading to negative outcomes such as increased risk of falls [22], hospitalization [23], need for long-term care [24], and mortality [25]. The global prevalence of frailty has risen with population aging, with an incidence rate in China reaching up to 60.6 per 1000 person-years [26]. Frailty has become a focal research topic within the aging research agenda in China during the 21st century [10]. Socio-demographic factors influence frailty risk, with rural residency identified as a risk factor [26]. Women are particularly vulnerable due to their disproportionate share of household and caregiving responsibilities, further elevating their likelihood of developing frailty [27].

However, limited studies examining frailty among informal caregivers have yielded inconsistent results. For instance, research from Brazil indicates that unpaid care was associated with higher odds of frailty [28]; findings from the US find no significant differences in frailty incidence between caregivers and non-caregivers [29]; a European study reveals that the effects of unpaid caregiving on frailty varied by gender and regions [30]. Research specifically on frailty among spousal caregivers remains scarce [31–33]. Existing studies relied on small sample sizes [31, 32], and focused primarily on dementia caregivers [33]. Importantly, all previous studies were conducted outside China; to the best of our knowledge, no research has examined this association within the Chinese population. Given China’s distinct cultural context and the profound implications of caregiver frailty—not only for caregivers’ health but also for caregiving sustainability—it is critical to investigate this relationship using a large, nationally representative Chinese sample.

Thus, this study aimed to examine this association between spousal caregiving and frailty, explore how this association varies with care intensity, and investigate how gender differences influence these relationships. Accordingly, the research question was: Does spousal caregiving influence caregivers’ frailty, and how does the extent of this effect vary with care intensity and gender? The following hypotheses were proposed:

H1: Spousal caregiving is associated with a higher frailty index than non-caregivers, with an accelerated rate of increase over time.

H2: The association between spousal caregiving and frailty is stronger among caregivers providing higher-intensity care than those providing lower- or medium-intensity care.

H3: The impact of spousal caregiving on frailty is particularly evident among female caregivers.

## Methods

### Data source and study population

Data were derived from the 2011, 2013, 2015 and 2018 waves of the China Health and Retirement Longitudinal Study (CHARLS), a nationally representative panel survey targeting middle-aged and older adults (45 years and above). CHARLS sampled 10,257 households across 150 counties in China, interviewing 17,708 individuals at baseline in 2011, with follow-up interviews conducted every two years.

In this study, participants were selected based on the following criteria: (i) both the main respondents and their spouses were interviewed at baseline and participated in subsequent follow-up waves; (ii) participants were aged 50 years or older at baseline; and (iii) participants completed the key survey sections related to spousal caregiving and frailty indicators across all four waves. Finally, this analysis included 3,987 participants, yielding a total of 15,948 observations. Cases with less than 2% missing data on covariates were directly excluded. However, for variables with substantial missing data, specifically current smoking status (1,086 missing cases) and financial support from children (386 missing cases), a missing indicator category was introduced. For detailed information on participant selection and missing data, see Appendix Figure S1 and Table S4.

### Measures

#### Frailty

The frailty index used in this study was developed and validated by Rockwood et al [34]. This approach calculates a frailty score by accumulating deficits in symptoms, signs, diseases, and disabilities to calculate a frailty score. The total reflects an individual’s likelihood of frailty, and typically, 30 to 40 deficits are recommended to accurately predict outcomes. In this study, frailty was assessed based on 41 indicators covering self-reported health, medically diagnosed conditions, medical symptoms, functional activities assessment, ADLs (activities of daily living), and IADLs (instrumental activities of daily living). The raw scores were calculated by summing the scores across 41 indicators. Mean frailty scores, used as continuous variables in our analysis, were obtained by dividing each total raw score by 41. For further details, see Appendix Table S1.

#### Caregiving status

Caregiving status was defined based on the respondent’s reported sources of assistance for ADLs and/or IADLs. Respondents identifying their spouse as their provider of these activities had their spouse classified as a spousal caregiver.

#### Care intensity

Care intensity was measured by assessing the care recipients’ level of dependency and was categorized into four groups: non-caregivers, lower intensity (only IADLs limitations), moderate intensity (1–3 ADLs limitations), and higher intensity (4–6 ADLs limitations).

#### Covariates

Covariates included both time-invariant and time-varying variables. The time-invariant variables were gender and education level. Time-varying variables included age, residence, working status, drinking and smoking status, depression, family size (number of the household members), financial support from children, medical insurance and the number of potential caregivers.

#### Statistical analysis

The analysis was performed using Stata version 18.0. First, a two-sample t-test and Chi-square test were conducted to evaluate the statistical significance of all covariates in relation to caregiving status. Subsequently, a growth curve model with random intercepts was used to examine the associations between repeated measures of caregiving status, care intensity, and frailty over the 7-year follow-up period. Potential gender differences were investigated using gender-stratified models. Since CHARLS participants were interviewed every two years, a time variable reflecting the chronological order of study waves was created and denoted as t = 0, 1, 2, 3, and subsequently incorporated into the models.

## Results

### Sample characteristics

Table 1 presents the characteristics of the 3,987 participants according to gender and caregiving status. Significant differences between caregivers and non-caregivers were observed for both females and males in terms of age, educational level, residence, depressive symptoms, financial support from children, family size, potential caregivers and frailty scores. However, among females, significant differences were also found in smoking and drinking status. Detailed comparisons between spousal caregivers and non-caregivers across the entire samples are provided in Appendix Table S2.

**Table 1:** Descriptive statistics of samples according to gender and caregiving status from waves 2011–2018 (N = 3987, observations = 15948)

	Overall (N = 3,987, obs = 15,948)	Females (N = 1,806, obs = 7,224)			Males (N = 2,181, obs = 8,724)		
Variables		Caregivers (obs = 1,042, 14.4%)	Non-caregivers (obs = 6,182, 85.6%)	*P Value*	Caregivers (obs = 1,762, 20.2%)	Non-caregivers (obs = 6,962, 79.8%)	*P Value*
Raw frailty scores (Mean, SD)	6.40(3.68)	8.46(4.32)	6.85(3.66)	*<0.001*	6.81(3.68)	5.59(3.37)	*<0.001*
Mean frailty scores (Mean, SD)	0.16(0.09)	0.21(0.11)	0.17(0.09)	*<0.001*	0.17(0.09)	0.14(0.08)	*<0.001*
Age (Mean, SD)	62.57(6.67)	61.16(6.72)	61.41(6.09)	*<0.001*	65.40(7.00)	62.64(6.81)	*<0.001*
Age group (N, %)				*<0.001*			*<0.001*
50-59	5,760(36.1)	283(27.2)	2,566(41.5)		376(21.3)	2,535(36.4)	
60-69	7,743(48.6)	545(52.3)	2,987(48.3)		917(52.0))	3,294(47.3)	
> = 70	2,445(15.3)	214(20.5)	629(10.2)		469(26.7)	1,133(16.3)	
Education (N, %)				*<0.001*			*<0.001*
less than lower secondary	14,120(88.5)	1,002(96.2)	5,766(93.3)		1,576(89.4)	5,776(83.0)	
upper secondary	1,828(11.5)	40(3.8)	416(6.7)		186(10.6)	1,186(17.0)	
Current working status (N, %)				*0.832*			*0.553*
no	4,907(30.8)	385(37.0)	2,263(36.6)		466(26.5)	1,793(25.8)	
yes	11,041(69.2))	657(63.0)	3,919(63.4)		1,296(73.5)	5,169(74.2)	
Residence (N, %)				*<0.001*			*<0.001*
rural	10,608(66.5)	766(73.5)	3,994(64.6)		440(25.0)	2,436(35.0)	
urban	5,340(33.5)	276(26.5)	2,188(35.4)		1,322(75.0)	4,526(65.0)	
Current drinking status (N, %)				*0.026*			*0.415*
no	10,012(62.8)	875(84.0)	5,350(86.5)		780(44.3)	3,007(43.2)	
yes	5,936(37.2)	167(16.0)	832(13.5)		982(55.7)	3,955(56.8)	
Current smoking status (N, %)				*<0.001*			*0.986*
no	10,443(65.5)	929(89.2)	5,843(94.5)		742(42.1)	2,929(41.1)	
yes	4,419(27.7)	96(9.2)	290(4.7)		816(46.3)	3,217(46.2)	
missing	1,086(6.8)	17(1.6)	49(0.8)		204(11.6)	816(11.7)	
Depression				*<0.001*			*<0.001*
no	10,824(67.9)	502(48.2)	3,885(62.8)		1,173(66.6)	5,264(75.6)	
depressive symptoms	4,269(26.8)	393(33.7)	1901(30.8)		476(27.0)	1,499(21.5)	
depression	855(5.3)	147(14.1)	396(6.4)		113(6.4)	199(2.9)	
Medical insure (N, %)				*0.822*			*0.840*
no	689(4.3)	49(4.7)	281(4.6)		71(4.0)	288(4.1)	
yes	15,259(95.7)	993(95.3)	5,901(95.5)		1,691(96.0)	6,674(95.9)	
Financial support from children (quintile, N, %)				*<0.001*			*<0.001*
1	5,326(33.4)	275(26.4)	2,100(34.0)		474(26.9)	2,477(35.6)	
2	2,949(18.5)	219(21.0)	1,116(18.1)		390(22.1)	1,224(17.6)	
3	3,423(21.5)	243(23.3)	1,314(21.3)		426(24.2)	1,440(20.7)	
4	3,864(24.2)	267(25.6)	1,506(24.3)		424(24.1)	1,667(23.9)	
missing	386(2.4)	38(3.7)	146(2.3)		48(2.7)	154(2.2)	
Family size (Mean, SD)	3.35(1.67)	3.21(1.60)	3.38(1.67)	*0.002*	3.12(1.57)	3.41(1.70)	*<0.001*
Potential caregivers (Mean, SD)	0.07(0.26)	0.24(0.42)	0.02(0.13)	*<0.001*	0.27(0.45)	0.05(0.21)	*<0.001*

*Notes:* CHARLS, China Health and Retirement Longitudinal Study; SD, standard deviation; N, number; %, percentage; obs, observations.

### Spousal caregiving and frailty trajectory

In the fully adjusted model for the entire sample, spousal caregivers exhibited a significantly higher frailty index (B = 0.009, SE = 0.003, *P* < .01) compared to non-caregivers, along with an accelerated rate of frailty increase over time (B = 0.002, SE = 0.001, *P* < .05).

When caregiving intensity was further examined, all three intensity levels—lower, moderate, and higher—were significantly associated with increased frailty across models (*P* < .05). In Model 7, both the lower-intensity (B = 0.003, SE = 0.002, *P* < .05) and moderate-intensity (B = 0.003, SE = 0.002, *P* < .10) showed an accelerated increase in frailty over time, as evidenced by significant or marginally significant interactions with the quadratic time term. However, these associations were attenuated after adjusting for depression. As shown in Model 8, the magnitude of the coefficients changed considerably, particularly for moderate-intensity (B = 0.008, SE = 0.004, *P* < .05) and higher-intensity caregivers (B = 0.017, SE = 0.006, *P* < .01). Detailed results were provided in Appendix Table S3 and Figure S2 to S3.

### Gender-difference in frailty trajectory

Among females (Table 2, Figure S4), findings were consistent with the overall sample results. Providing spousal care was significantly associated with a higher frailty index (B = 0.016, SE = 0.004, *P* < .001) after controlling for all covariates. Although female caregivers initially showed slower frailty progression (B = −0.014, SE = 0.006, *P* < .05), their frailty increased at a faster rate over time (B = 0.005, SE = 0.002, *P* < .01). Conversely, among males (Table 3, Figure S6), spousal caregiving was not significantly associated with frailty trajectory (B = 0.003, SE = 0.003, *P* > .05).

**Table 2:** Longitudinal association of caregiving and frailty among females (N = 1806, observations = 7224)

Frailty								
	Model1	Model2	Model3	Model4	Model5	Model6	Model7	Model8
Care status								
Intercept	0.141^***^ (0.002)	0.145^***^ (0.002)	0.146^***^ (0.002)	0.145^***^ (0.002)	0.145^***^ (0.004)	0.145^***^ (0.004)	0.142^***^ (0.005)	0.131^***^ (0.005)
Non-caregivers as ref.								
Caregiver	0.017^***^ (0.002)	0.017^***^ (0.002)	0.013^**^ (0.004)	0.021^***^ (0.004)	0.021^***^ (0.004)	0.021^***^ (0.004)	0.020^***^ (0.004)	0.016^***^ (0.004)
Time	0.020^***^ (0.001)	0.005^**^ (0.002)	0.005^**^ (0.002)	0.008^***^ (0.002)	0.008^***^ (0.002)	0.007^***^ (0.002)	0.006^***^ (0.002)	0.008^***^ (0.002)
Time^*^time		0.005^***^ (0.001)	0.005^***^ (0.001)	0.004^***^ (0.001)	0.004^***^ (0.001)	0.004^***^ (0.001)	0.004^***^ (0.001)	0.003^***^ (0.001)
Non-caregivers as ref.								
Caregiver^*^time			0.002 (0.002)	−0.017^**^ (0.006)	−0.017^**^ (0.006)	−0.017^**^ (0.006)	−0.017^**^ (0.006)	−0.014^*^ (0.006)
Caregiver^*^time^*^time				0.006^**^ (0.002)	0.006^**^ (0.002)	0.006^**^ (0.002)	0.006^**^ (0.002)	0.005^**^ (0.002)
Care intensity								
Intercept	0.141^***^ (0.002)	0.145^***^ (0.002)	0.146^***^ (0.002)	0.145^***^ (0.002)	0.145^***^ (0.004)	0.145^***^ (0.004)	0.142^***^ (0.005)	0.131^***^ (0.005)
Non-caregivers as ref.								
Lower intensity	0.012^***^ (0.003)	0.013^***^ (0.003)	0.012^*^ (0.005)	0.022^**^ (0.007)	0.022^**^ (0.007)	0.022^**^ (0.007)	0.021^**^ (0.007)	0.018^**^ (0.006)
Moderate intensity	0.021^***^ (0.003)	0.021^***^ (0.003)	0.015^**^ (0.006)	0.020^**^ (0.007)	0.019^**^ (0.007)	0.019^**^ (0.007)	0.018^**^ (0.007)	0.015^*^ (0.006)
Higher intensity	0.027^***^ (0.005)	0.026^***^ (0.005)	0.022^*^ (0.010)	0.032^**^ (0.012)	0.031^**^ (0.011)	0.030^**^ (0.011)	0.029^**^ (0.011)	0.017 (0.011)
Time	0.020^***^ (0.001)	0.006^**^ (0.002)	0.006^**^ (0.002)	0.008^***^ (0.002)	0.008^***^ (0.002)	0.007^***^ (0.002)	0.006^**^ (0.002)	0.008^***^ (0.002)
Time^*^time		0.005^***^ (0.001)	0.005^***^ (0.001)	0.004^***^ (0.001)	0.004^***^ (0.001)	0.004^***^ (0.001)	0.004^***^ (0.001)	0.003^***^ (0.001)
Non-caregivers as ref.								
Care intensity^*^time								
Lower intensity			0.001 (0.003)	−0.021^*^ (0.009)	−0.021^*^ (0.009)	−0.021^*^ (0.009)	−0.022^*^ (0.009)	−0.019^*^ (0.009)
Moderate intensity			0.003 (0.003)	−0.010 (0.009)	−0.010 (0.009)	−0.010 (0.009)	−0.010 (0.009)	−0.011 (0.009)
Higher intensity			0.002 (0.004)	−0.020 (0.015)	−0.020 (0.015)	−0.020 (0.015)	−0.019 (0.015)	−0.009 (0.014)
Care intensity^*^time^*^time								
Lower intensity				0.007^*^ (0.003)	0.007^*^ (0.003)	0.007^*^ (0.003)	0.007^*^ (0.003)	0.006^*^ (0.003)
Moderate intensity				0.004 (0.003)	0.004 (0.003)	0.004 (0.003)	0.004 (0.003)	0.004 (0.003)
Higher intensity				0.007 (0.004)	0.007 (0.004)	0.007 (0.004)	0.006 (0.004)	0.003 (0.004)

*Notes:* Model 1: Caregiving status and time; Model 2: Model 1 + quadratic term for time; Model 3: Model 2 + interaction between caregiving status and time; Model 4: Model 3 + interaction between caregiving status and the quadratic term for time; Model 5: Model 4 + age, gender, education level, residence, and current working status; Model 6: Model 5 + current drinking and smoking status; Model 7: Model 6 + medical insurance, financial support from children, number of potential caregivers, and family size; Model 8 (fully adjusted model): Model 7 + depression.

^+^ *P* < .10, ^*^P < .05, ^**^P < .01, ^***^P < .001.

**Table 3:** Longitudinal association of caregiving and frailty among males (N = 2,181, observations = 8,724)

Frailty								
	Model1	Model2	Model3	Model4	Model5	Model6	Model7	Model8
Care status								
Intercept	0.115^***^ (0.002)	0.120^***^ (0.002)	0.121^**^ (0.002)	0.120^***^ (0.002)	0.126^***^ (0.003)	0.138^***^ (0.003)	0.138^***^ (0.005)	0.131^***^ (0.004)
Non-caregivers as ref.								
Caregiver	0.007^***^ (0.002)	0.008^***^ (0.002)	0.005^+^ (0.003)	0.006^+^ (0.003)	0.006^*^ (0.003)	0.007^*^ (0.003)	0.005^*^ (0.003)	0.003 (0.003)
Time	0.017^***^ (0.000)	0.001 (0.001)	0.001 (0.001)	0.001 (0.002)	0.001 (0.002)	0.001 (0.002)	−0.000 (0.002)	0.001 (0.002)
Time^*^time		0.005^***^ (0.000)	0.006^***^ (0.000)	0.005^***^ (0.001)	0.005^***^ (0.001)	0.005^***^ (0.001)	0.005^***^ (0.001)	0.004^***^ (0.001)
Non-caregivers as ref.								
Caregiver^*^time			0.002^+^ (0.001)	−0.001 (0.004)	−0.002 (0.004)	−0.001 (0.004)	−0.002 (0.004)	0.000 (0.004)
Caregiver^*^time^*^time				0.001 (0.001)	0.001 (0.001)	0.001 (0.001)	0.001 (0.001)	0.001 (0.001)
Care intensity								
Intercept	0.115^***^ (0.002)	0.120^***^ (0.002)	0.120^***^ (0.002)	0.120^***^ (0.002)	0.126^***^ (0.003)	0.137^***^ (0.003)	0.138^***^ (0.005)	0.131^***^ (0.004)
Non-caregivers as ref.								
Lower intensity	0.004^*^ (0.002)	0.006^**^ (0.002)	0.001 (0.003)	0.002 (0.004)	0.003 (0.004)	0.003 (0.004)	0.001 (0.004)	0.000 (0.004)
Moderate intensity	0.009^***^ (0.002)	0.010^***^ (0.002)	0.004 (0.004)	0.006 (0.005)	0.006 (0.005)	0.007 (0.005)	0.006 (0.005)	0.003 (0.005)
Higher intensity	0.022^***^ (0.004)	0.021^***^ (0.004)	0.028^***^ (0.007)	0.022^**^ (0.008)	0.023^**^ (0.008)	0.024^**^ (0.008)	0.023^**^ (0.008)	0.015^*^ (0.008)
Time	0.017^***^ (0.000)	0.001 (0.001)	0.001 (0.001)	0.001 (0.002)	0.001 (0.002)	0.001 (0.002)	−0.000 (0.002)	0.001 (0.002)
Time^*^time		0.005^***^ (0.000)	0.005^***^ (0.000)	0.005^**^ (0.001)	0.005^***^ (0.001)	0.005^***^ (0.001)	0.005^***^ (0.001)	0.004^***^ (0.001)
Non-caregivers as ref.								
Care intensity^*^time								
Lower intensity			0.003 (0.002)	0.000 (0.006)	−0.001 (0.006)	−0.000 (0.006)	−0.001 (0.006)	0.000 (0.006)
Moderate intensity			0.003^+^ (0.002)	−0.001 (0.006)	−0.002 (0.006)	−0.002 (0.006)	−0.003 (0.006)	−0.000 (0.006)
Higher intensity			−0.004 (0.003)	0.012 (0.011)	0.009 (0.011)	0.008 (0.011)	0.007 (0.011)	0.010 (0.011)
Care intensity^*^time^*^time								
Lower intensity				0.001 (0.002)	0.001 (0.002)	0.001 (0.002)	0.001 (0.002)	0.001 (0.002)
Moderate intensity				0.001 (0.002)	0.002 (0.002)	0.002 (0.002)	0.002 (0.002)	0.001 (0.0032
Higher intensity				−0.005 (0.003)	−0.004 (0.003)	−0.004 (0.003)	−0.004 (0.003)	−0.004 (0.003)

*Notes:* Model 1: Caregiving status and time; Model 2: Model 1 + quadratic term for time; Model 3: Model 2 + interaction between caregiving status and time; Model 4: Model 3 + interaction between caregiving status and the quadratic term for time; Model 5: Model 4 + age, gender, education level, residence, and current working status; Model 6: Model 5 + current drinking and smoking status; Model 7: Model 6 + medical insurance, financial support from children, number of potential caregivers, and family size; Model8 (fully adjusted model): Model 7 + depression.

^+^ *P* < .10, ^*^P < .05, ^**^P < .01, ^***^P < .001.

Regarding care intensity among females (Table 2, Figure S5), lower- (B = 0.021, SE = 0.007, *P* < .01), moderate- (B = 0.018, SE = 0.007, *P* < .01), and higher-intensity (B = 0.029, SE = 0.011, *P* < .01) caregivers had significantly higher frailty indices compared to female non-caregivers in model unadjusted for depression. However, after controlling for depression, the significant effect among higher-intensity caregivers was attenuated (B = 0.017, SE = 0.011, *P* > .10). Additionally, frailty increased at an accelerated rate among female caregivers providing lower-intensity care (B = 0.006, SE = 0.003, *P* < .05).

Among male caregivers (Table 3, Figure S7), only those providing higher-intensity care demonstrated a significantly higher frailty index care compared to non-caregiving males (B = 0.015, SE = 0.008, *P* < .05).

## Discussion

This is the first study to examine the association between spousal caregiving status, and frailty trajectories over a seven-year period in China, explore how this association varies with care intensity, and investigate how gender differences influence these relationships. Our findings support the proposed hypotheses and highlight the importance of developing targeted support systems for spousal caregivers, particularly female and higher-intensity male caregivers.

Our study is novel and extends existing evidence, partially aligning with findings from other countries [31–33], which suggest that spousal caregivers had higher frailty indices than non-caregivers and experienced faster frailty progression over time. The strength of this association varied by care intensity, with the strongest effects observed among caregivers engaged in higher-intensity care. Similar findings in China have indicated that spousal caregivers sacrificed their own health while providing care [11–15, 19].

Several explanations could explain this relationship. First, spousal caregiving typically occurs within the co-residential setting, where caregivers often assume extensive responsibilities beyond basic ADLs/IADLs assistance, increasing the caregiving burden. Assisting a spouse with ADLs limitations entails greater physical [12], emotional [13] and cognitive [15] stress than assisting those with only IADLs limitations. According to the stress and coping model [35], prolonged caregiving acts as a chronic stressor, thereby contributing to frailty. Supporting this, findings from the Survey of Health, Aging and Retirement in Europe (SHARE) [30] also report increased frailty risk among co-residential caregivers.

Spousal caregivers also face reduced leisure time due to the demanding and unpaid nature of caregiving, aligning with time poverty theory [36]. Originally developed to describe households where adults lacked sufficient working hours to avoid poverty, the theory has since expanded to include a broader impact on well-being. The discretionary time theory further emphasizes the critical role of leisure time—after essential work and personal care—for maintaining health [37]. Given that spousal caregiving reduces time for social engagement and physical activities—both known protective factors against frailty—caregivers are more likely to experience health deterioration [38].

Cultural perspectives in China also influence caregiving dynamics. While caregiving for children and/or grandchildren is framed as nurturing ‘the hope of the future’ and investing in ‘prospective caregivers for themselves’, caregiving for parents is grounded in filial piety, and spousal caregiving is often viewed less as a voluntary act and more as a moral obligation. Although Confucianism [5] promotes mutual spousal support, what is provided between spouses is frequently described as a ‘labour of love’ [39], shaped by emotional closeness rather than reciprocal duty. When caregiving is perceived as inevitable rather than chosen, psychological distress and chronic stress can intensify caregivers’ vulnerability to frailty [40].

Gender differences in the impact of spousal caregiving on frailty reveal a stronger effect among females, whereas among male caregivers, only those providing higher-intensity care exhibited significantly higher frailty indices compared to their non-caregiving counterparts. These findings partially align with European research [30], and are consistent with Chinese studies showing that female caregivers were susceptible to adverse health outcomes [15, 18, 19].

Several factors explain this gender disparity. When husbands require assistance, wives typically assume primary caregiving responsibilities, often with limited or secondary support. Even when other potential caregivers are available, wives tend to carry the burden, a reflection of cultural gender norms [16]. Conversely, male caregivers are more likely to receive assistance [41], and may delegate intimate caregiving tasks, such as bathing and incontinence management, to adult daughters, or social workers due to cultural discomfort. As caregiving is socially constructed as an extension of women’s household roles, female caregivers often experience increasing and compounding workloads over time [42]. Moreover, husbands typically engage extensively in caregiving only when their wives had a formal diagnosis of illness or significant care needs [43]. In such scenarios, female spousal caregivers tend to assume broader caregiving duties, increasing their caregiving burden and elevating their frailty risk.

In addition, female caregivers often report fewer positive emotional experiences than males [44, 45]. Women are typically socialized to view caregiving as a duty rather than an act of voluntary support, and routine caregiving tasks—such as meal preparation, household chores, grocery shopping, and medication management—are often taken for granted by spouses. In contrast, male caregivers receive more recognition, which fosters a sense of personal growth, achievement, and purpose. These positive experiences have been linked to a reduced risk of frailty among male caregivers [46].

Various unobserved factors—including physiological, biological, epigenetic, and psychological mechanisms—also contribute to gender disparities in frailty [47]. Menopause-related declines in oestrogen among females contribute to decreased bone mass density and heightened risk of osteoporosis, conditions linked to increased frailty [48]. Genetic differences—such as the presence of two X chromosomes in females containing inflammation-related genes—heighten systemic inflammation [49], and contributed to frailty risk [50]. Furthermore, psychological stress responses and coping mechanisms also vary by gender. Females tend to experience higher stress levels and adopt passive, emotion-focused coping strategies, whereas males favour active, problem-solving approaches [51]. In the context of spousal caregiving, passive coping and reduced support-seeking among females may result in prolonged psychological distress, increasing their vulnerability to chronic stress and frailty [40].

While these mechanisms help explain gender disparities, higher-intensity caregiving also significantly increased frailty risk among male caregivers compared to their non-caregiving counterparts. As previously mentioned [43], when wives have substantial care needs, husbands become sole caregivers, assuming full responsibilities, which may contribute to frailty.

When examining care intensity among females, our findings indicate that even those providing lower-intensity care (only IADLs) were associated with higher frailty. This contrasts with previous research on depression [18] and cognition [15], which report that providing IADLs assistance was not associated with increased depression [18] or significantly change rate of cognition when compared to males [15]. This divergence may be attributed to differences in outcome measures. Frailty, as a multidimensional construct, may be more sensitive to the caregiving burden.

Furthermore, depression played a critical role in the association between higher-intensity care and frailty among female caregivers, as adjusting for depression attenuated this association. Prior research confirms that depressive symptoms were closely linked to increased frailty among older adults [52], and that higher positive affect among female caregivers was protective [29]. These findings emphasize the importance of psychological well-being in mitigating adverse health outcomes associated with spousal caregiving, particularly among females.

## Limitations

Our study has several dataset-related limitations. First, as most variables were self-reported, recall bias may have influenced our findings. Second, due to a system error in CHARLS, information on total caregiving hours per day and total caregiving days per month was unavailable in the 2013 wave, precluding the use of caregiving time as a measure of caregiving intensity. Third, the 2013 questionnaire design did not allow us to specify the type of limitations—IADLs or ADLs—for which spousal caregivers provided assistance. Although we assessed caregiving intensity, our evaluation depended entirely on care recipients’ self-reported ADLs/IADLs limitations. For instance, if a care recipient reported limitations in both domains and indicated receiving care from their spouse, we assumed that the spouse provided care in both domains, potentially introducing bias. Fourth, most participants were from rural areas, limiting generalizability. Lastly, certain potential confounders, such as physical activity and nutrition, were excluded due to CHARLS study design constraints. For example, in CHARLS, physical activity questions were randomly sampled, and nutritional measures available in CHARLS were limited.

## Conclusions

This study demonstrates that female spousal caregiving was associated with an increased frailty risk, with the rate of frailty progression accelerating over time among those providing lower-intensity care. Depression plays a key role in these associations. Additionally, male caregivers providing higher-intensity care also exhibited a higher frailty index compared to their counterparts. These findings highlight the need to implement targeted supportive measures to reduce psychological stress and mitigate the long-term health risks associated with spousal caregiving.

## Supplementary Material

aa-24-2705-File002_afaf148

## Research Data Transparency and Availability

The dataset analysed during this current study are available from the China Health and Retirement Longitudinal study (CHARLS), waves 2011, 2013, 2015 and 2018, https://charls.charlsdata.com/pages/data/111/en.html.

## References

[1] World Health Organization (WHO). Global Strategy and Action Plan on Ageing and Health. Geneva: World Health Organization, 2017.

[2] Rudnicka E, Napierała P, Podfigurna A et al. The World Health Organization (WHO) approach to healthy ageing. Maturitas 2020; 139: 6–11. 10.1016/j.maturitas.2020.05.018. 32747042 PMC7250103

[3] Addati L, Cattaneo U, Esquivel V et al. Care Work and Care Jobs for the Future of Decent Work. Geneva: International Labour Organization, 2018.

[4] World Health Organization(WHO) . China Country Assessment Report on Ageing and Health. Geneva: World Health Organization, 2015.

[5] Slote WH, DeVos GA. In: Slote WH, De Vos GA, eds. Confucianism and the Family. Albany, N.Y: State University of New York Press, 1998.

[6] Chen X, Giles J, Yao Y et al. The path to healthy ageing in China: a Peking University–lancet commission. The Lancet (British edition) 2022; 400: 1967–2006. 10.1016/S0140-6736(22)01546-X. PMC9801271 36423650

[7] Liu X, Lu B, Feng Z. Intergenerational transfers and informal care for disabled elderly persons in China: evidence from CHARLS. Health Soc Care Community 2017; 25: 1364–74. 10.1111/hsc.12441. 28276169

[8] Yang S, Chen W. Changes in family structure in China: the impact of residence patterns and demographic factors. China Population and Development Studies 2019 2:4 2019; 2: 401–11.

[9] Li M, Dai H. Determining the primary caregiver for disabled older adults in mainland China: spouse priority and living arrangements. J Fam Ther 2019; 41: 126–41. 10.1111/1467-6427.12213.

[10] National Health and Family Planning Commission of the People’s Republic of China . Report on the Family Development in China. Beijing: China Population and Development Research Center, 2015.

[11] Wang R . The influence of spousal care on the health of middle-aged and older adult caregivers in China—empirical analysis based on CHARLS data. Front Public Health 2025; 13: 1496637. 10.3389/fpubh.2025.1496637. 39980908 PMC11839437

[12] Liu H, Lou VWQ. Transitioning into spousal caregiving: contribution of caregiving intensity and caregivers’ multiple chronic conditions to functional health. Age Ageing 2019; 48: 108–14. 10.1093/ageing/afy098. 29982336

[13] Ma J, Yang H, Hu W et al. Spousal care intensity, socioeconomic status, and depression among the older caregivers in China: a study on 2011–2018 CHARLS panel data. Healthcare (Basel) 2022; 10: 239. 10.3390/healthcare10020239. 35206854 PMC8872002

[14] Zhao X, Liu H, Fang B et al. Continuous participation in social activities as a protective factor against depressive symptoms among older adults who started high-intensity spousal caregiving: findings from the China health and retirement longitudinal survey. Aging Ment Health 2021; 25: 1821–9. 10.1080/13607863.2020.1822283. 32954798

[15] Guo Y, Zhang Z, Jiang Q. Spousal caregiving types and cognitive trajectories among middle-aged and older adults in China. Res Aging 2025; 47: 308–20. 10.1177/01640275251317544. 39895081

[16] Holroyd E . Developing a cultural model of caregiving obligations for elderly Chinese wives. West J Nurs Res 2005; 27: 437–56. 10.1177/0193945905274907. 15870240

[17] Ngan R, Wong W. Injustice in family Care of the Chinese Elderly in Hong Kong. J Aging Soc Policy 1996; 7: 77–94. 10.1300/J031v07n02_06. 10183216

[18] Duan H, Chen F. Gender, spousal caregiving, and depressive symptoms among Chinese older adults: does work status matter? Aging Ment Health 2023; 27: 124–32. 10.1080/13607863.2022.2032596. 35109739

[19] Ai J, Feng J, Yu Y. Elderly care provision and the impact on caregiver health in China. Chin World Econ 2022; 30: 206–26. 10.1111/cwe.12443.

[20] Epel ES, Crosswell AD, Mayer SE et al. More than a feeling: a unified view of stress measurement for population science. Front Neuroendocrinol 2018; 49: 146–69. 10.1016/j.yfrne.2018.03.001. 29551356 PMC6345505

[21] Fedarko NS . The biology of aging and frailty. Clin Geriatr Med 2011; 27: 27–37. 10.1016/j.cger.2010.08.006. 21093720 PMC3052959

[22] Cheng M-H, Chang S-F. Frailty as a risk factor for falls among community dwelling people: evidence from a meta-analysis. J Nurs Scholarsh 2017; 49: 529–36. 10.1111/jnu.12322. 28755453

[23] Chang S-F, Lin H-C, Cheng C-L. The relationship of frailty and hospitalization among older people: evidence from a meta-analysis. J Nurs Scholarsh 2018; 50: 383–91. 10.1111/jnu.12397. 29874399

[24] Chen S, Honda T, Narazaki K et al. Physical frailty and risk of needing long-term Care in Community-Dwelling Older Adults: a 6-year prospective study in Japan. J Nutr Health Aging 2019; 23: 856–61. 10.1007/s12603-019-1242-6.31641736

[25] Kojima G, Iliffe S, Walters K. Frailty index as a predictor of mortality: a systematic review and meta-analysis. Age Ageing 2018; 47: 193–200. 10.1093/ageing/afx162. 29040347

[26] Xu W, Li YX, Wu C. Incidence of frailty among community-dwelling older adults: a nationally representative profile in China. BMC Geriatr 2019; 19: 378–8. 10.1186/s12877-019-1393-7. 31888498 PMC6937935

[27] Wang HY, Zhang M, Sun X. Sex-specific association between socioeconomic status, lifestyle, and the risk of frailty among the elderly in China. Front Med (Lausanne) 2021; 8: 775518. 10.3389/fmed.2021.775518. 34869494 PMC8639189

[28] dos Santos-Orlandi AA, Brigola AG, Ottaviani AC et al. Elderly caregivers of the elderly: frailty, loneliness and depressive symptoms. Rev Bras Enferm 2019; 72: 88–96. 10.1590/0034-7167-2018-0137. 31826196

[29] Park-Lee E, Fredman L, Hochberg M et al. Positive affect and incidence of frailty in elderly women caregivers and noncaregivers: results of caregiver-study of osteoporotic fractures. Journal of the American Geriatrics Society (JAGS) 2009; 57: 627–33. 10.1111/j.1532-5415.2009.02183.x. PMC3711829 19392954

[30] Barbosa F, Voss G, Delerue MA. Do European co-residential caregivers aged 50+ have an increased risk of frailty? Health Soc Care Community 2020; 28: 2418–30. 10.1111/hsc.13064. 32557977 PMC7818189

[31] Potier F, Degryse J-M, Bihin B et al. Health and frailty among older spousal caregivers: an observational cohort study in Belgium. BMC Geriatr 2018; 18: 291. 10.1186/s12877-018-0980-3. 30477431 PMC6258488

[32] Potier F, Degryse J-M, Aubouy G et al. Spousal caregiving is associated with an increased risk of frailty: a case-control study. J Frailty Aging 2018; 7: 170–5. 10.14283/jfa.2018.11. 30095147

[33] Dassel KB, Carr DC. Does dementia caregiving accelerate frailty? Findings from the health and retirement study. Gerontologist 2016; 56: 444–50. 10.1093/geront/gnu078. 25161263

[34] Rockwood K, Mitnitski A. Frailty in relation to the accumulation of deficits. J Gerontol A Biol Sci Med Sci 2007; 62: 722–7. 10.1093/gerona/62.7.722. 17634318

[35] Lazarus RS . In: Susan F, ed. Stress, Appraisal, and Coping. New York: Springer, 1984.

[36] Vickery C . The time-poor: a new look at poverty. J Hum Resour 1977; 12: 27–48. 10.2307/145597.

[37] Williams JR, Masuda YJ, Tallis H. A measure whose time has come. Soc Indic Res 2016; 128: 265–83. 10.1007/s11205-015-1029-z.

[38] Wang Y, Chen Z, Zhou C. Social engagement and physical frailty in later life: does marital status matter? BMC Geriatr 2021; 21: 248–8. 10.1186/s12877-021-02194-x. 33858354 PMC8047563

[39] Janet F, Dulcie G. In: Janet F, Dulcie G, eds. A Labour of Love: Women, Work, and Caring. London: Routledge & K. Paul, 1983.

[40] Lee SH, Shin J, Um S et al. Perceived stress and frailty in older adults. Ann Geriatr Med Res 2023; 27: 310–4. 10.4235/agmr.23.0132. 37704370 PMC10772339

[41] Feld S, Dunkle RE, Schroepfer T et al. Does gender moderate factors associated with whether spouses are the sole providers of IADL care to their partners? Res Aging 2010; 32: 499–526. 10.1177/0164027510361461. 21818168 PMC3148766

[42] Calasanti T, Bowen ME. Spousal caregiving and crossing gender boundaries: maintaining gendered identities. J Aging Stud 2006; 20: 253–63. 10.1016/j.jaging.2005.08.001.

[43] Langner LA, Furstenberg FF. Gender differences in spousal caregivers’ care and housework: fact or fiction? J Gerontol B Psychol Sci Soc Sci 2020; 75: 173–83. 10.1093/geronb/gby087. 30085145

[44] Kim Y, Baker F, Spillers RL. Cancer caregivers’ quality of life: effects of gender, relationship, and appraisal. J Pain Symptom Manage 2007; 34: 294–304. 10.1016/j.jpainsymman.2006.11.012. 17572056

[45] Wong DFK, Ng TK, Zhuang XY. Caregiving burden and psychological distress in Chinese spousal caregivers: gender difference in the moderating role of positive aspects of caregiving. Aging Ment Health 2019; 23: 976–83. 10.1080/13607863.2018.1474447. 29781713

[46] Wennberg AM, Anderson LR, Chen-Edinboro LP et al. Positive aspects of caregiving are associated with lower risk of frailty and sleep disruption in the National Study of caregiving. Innov Aging 2022; 6: igac058. 10.1093/geroni/igac058. 36267323 PMC9579720

[47] Zeidan RS, McElroy T, Rathor L et al. Sex differences in frailty among older adults. Exp Gerontol 2023; 184: 112333–3. 10.1016/j.exger.2023.112333. 37993077

[48] Ji M, Yu Q. Primary osteoporosis in postmenopausal women. Maturitas 2015; 82: 315–5. 10.1016/j.maturitas.2015.06.009. PMC5643776 29062981

[49] Casimir GJA, Duchateau J. Gender differences in inflammatory processes could explain poorer prognosis for males. J Clin Microbiol 2011; 49: 478–8, 9. 10.1128/JCM.02096-10. 21193776 PMC3020457

[50] Pothier K, Gana W, Bailly N et al. Associations between frailty and inflammation, physical, and psycho-social health in older adults: a systematic review. Front Psychol 2022; 13: 805501–1. 10.3389/fpsyg.2022.805501. 35360636 PMC8963891

[51] Matud MP . Gender differences in stress and coping styles. Pers Individ Dif 2004; 37: 1401–15. 10.1016/j.paid.2004.01.010.

[52] Zhou J, Chen H, Lin C. Frailty in the elderly is associated with an increased risk of depression: a systematic review and meta-analysis. Alpha Psychiatry 2024; 25: 175–82. 10.5152/alphapsychiatry.2024.231362. 38798812 PMC11117430

