# Supplementary material for: Impact of spousal caregiving on frailty index: longitudinal evidence from China Health and Retirement Longitudinal Study
Source: Age Ageing. 2025 Jun 8;54(6):afaf148. doi: 10.1093/ageing/afaf148 (PMC12145876; doi:10.1093/ageing/afaf148)
Supplement: aa-24-2705-File002_afaf148 [file aa-24-2705-file002_afaf148.docx]

**Supplementary file:**

**Impact of spousal caregiving on frailty index: Longitudinal Evidence from** **China Health and Retirement Longitudinal Study**

**Tables and figures of list:**

**Figure S 1.** Flow chart of selection process of participants in this study

**Table S 1.** Frailty index in this study

**Table S 2.** Descriptive statistics by caregiving status for spousal sample from CHARLS from 2011 to 2018 (N=3,987, Observations=15,948)

**Table S 3.** Longitudinal association of caregiving and frailty among the whole sample (N=3,987, observations=15,948)

**Table S 4.** Proportion of missingness across four waves (N=4,143, excluding outcome missing)

**Figure S 2.** Trajectory of frailty index by caregiving status among the whole sample

**Figure S 3.** Trajectory of frailty index by caregiving intensity among the whole sample

**Figure S 4.** Trajectory of frailty index by caregiving status among females

**Figure S 5.** Trajectory of frailty index by caregiving intensity among females

**Figure S 6.** Trajectory of frailty index by caregiving status among males

**Figure S 7.** Trajectory of frailty index by caregiving intensity among males

**Table S 4.** Proportion of missingness across four waves (N=4,143, excluding outcome missing)

**Figure S 1.** Flow chart of selection process of participants in this study

8,663 participants with clear caregiving status

8,670 participants across four waves and with a spouse

7 (care status not clear)

6,668 participants aged 50 years and over

1,995 (baseline age <50)

6,650 participants involved in outcome part

18 (not participated in frailty index part)

4,143 participants with complete outcome

2,507(frailty missing)

3,987 participants with complete data

155(work, drink, depression, medical insurance missing)

Wave 2013:

Caregivers: 725

Non-caregivers: 3,262

Wave 2011:

Caregivers: 429

Non-caregivers: 3,558

Wave 2015:

Caregivers: 780

Non-caregivers: 3,207

Wave 2018:

Caregivers: 870

Non-caregivers: 3,117

For binary variables, responses were coded 0 (“no”), and 1 (“yes”). Four-level categorical ordinal variables, such as ADLs, were scored as follows: “No, I do not have any difficulty” (0 points), “I have difficulty but can still do it” (0.33 points), “Yes, I have difficulty and need help” (0.67 points), and “I cannot do it” (1 point). Five-level categorical ordinal variables, such as self-reported health, were scored as: “Very good” (0 points), “Good” (0.25 points), “Fair” (0.5 points), “Poor” (0.75 points), and “Very poor” (1 point). ***See Table S1.***

**Table S 1.** Frailty index in this study

| **Domain** | **Number of indices** | **Variables in CHARLS** | **Coding of variables** |
| --- | --- | --- | --- |
| **Self-reported health** | 1 | Would you say your health is very good, good, fair, poor or very poor? | Very good=0  Good=0.25  Fair=0.5  Poor=0.75  Very poor=1 |
| **Medically diagnosed conditions** | 14 | Have you been diagnosed with the following diseases by a doctor?  Hypertension; Dyslipidemia; Diabetes or high blood sugar; Cancer or malignant tumor; Chronic lung diseases; Liver disease; Heart problems; Stroke; Kidney disease; Stomach or other digestive disease; Emotional, nervous, or psychiatric problems; Memory-related disease; Arthritis or rheumatism; Asthma | No=0  Yes=1 |
| **Medical symptoms** | 6 | - Have you ever fractured your hip? (Yes/No) - How good is your eyesight for seeing things at a distance, like recognizing a friend from across the street (with glasses or corrective lenses if you wear them)? Would you say your eyesight for seeing things at a distance is excellent, very good, good, fair, or poor? - How good is your eyesight for seeing things up close, like reading ordinary newspaper print (with glasses or corrective lenses if you wear them)? Would you say your eyesight for seeing things up close is excellent, very good, good, fair, or poor? - Is your hearing very good, good, fair, poor, or very poor (with a hearing aid if you normally use it and without if you normally don’t)? Would you say your hearing is excellent, very good, good, fair, or poor? - Have you lost all of your teeth? (Yes/No) - Are you often troubled with any body pains (Yes/No) | Yes=1  No=0  Excellent=0  Good=0.25  Fair=0.5  Poor=0.75  Very poor=1 |
| **Functional activities assessment** | 9 | Do you have any difficulty with   - running or jogging about 1 Km, - walking 1 km, - walking 100 meters, - getting up from a chair after sitting for a long period, - climbing several flights of stairs without resting, - stooping, kneeling, or crouching, reaching or extending arms above shoulder level, - lifting or carrying weights over 5 kg, - picking up a small coin from a table | “No, I don’t have any difficulty” =0  “I have difficulty but can still do it” =0.33  “Yes, I have difficulty and need help” =0.67  “I can not do it” =1 |
| **Activities of Daily Living (ADLs) and Instrumental Activities of Daily Living (IADLs)** | 11 | Do you have any difficulties with these because of a physical, mental, emotional or memory problem?   - dressing, - bathing or showering, - eating, - getting into or out of bed, - using the toilet, - controlling urination and defecation, - doing household chores, - preparing hot meals, - shopping for groceries, - managing money, - taking medications | “No, I don’t have any difficulty” =0  “I have difficulty but can still do it” =0.33  “Yes, I have difficulty and need help” =0.67  “I can not do it” =1 |

*Notes:* CHARLS, China Health and Retirement Longitudinal Study; KG, kilogram; KM, kilometer.

**Table S 2.** Descriptive statistics by caregiving status for spousal sample from CHARLS from 2011 to 2018 (N=3,987, Observations=15,948)

| Variables |  | Overall (N=3,987, obs=15,948) | Caregivers (N=701, obs=2,804, 17.6%) | Non-caregivers (N=3,286, obs=13,144 82.4%) | *P Value* |
| --- | --- | --- | --- | --- | --- |
| Raw frailty (Mean, SD) |  | 6.40(3.68) | 7.42(4.01) | 6.18(3.57) | *<0.001* |
| Mean frailty (Mean, SD) |  | 0.16(0.09) | 0.18(0.10) | 0.15(0.09) | *<0.001* |
| Age (Mean, SD) |  | 62.57(6.67) | 64.94(6.92) | 62.06(6.51) | *<0.001* |
| Age group (N, %) |  |  |  |  | *<0.001* |
|  | 50-59 | 5,760(36.1) | 659(23.6) | 5,101(38.8) |  |
|  | 60-69 | 7,743(48.6) | 1,462(52.1) | 6,281(44.8) |  |
|  | >=70 | 2,445(15.3) | 683(24.3) | 1,762(13.4) |  |
| Gender (N, %) |  |  |  |  | *<0.001* |
|  | female | 7,224(45.3) | 1,042(37.2) | 6,182(47.0) |  |
|  | male | 8,724(54.7) | 1,762(62.8) | 6,962(53.0) |  |
| Education (N, %) |  |  |  |  | *<0.001* |
|  | less than lower secondary | 14,120(88.5) | 2,578(91.9) | 11,542(87.8) |  |
|  | upper secondary, vocational training and tertiary | 1,828(11.5) | 226(8.1) | 1,602(12.2) |  |
| Current working status (N, %) |  |  |  |  | *0.596* |
|  | no | 4,907(30.8) | 851(30.4) | 4,05630.9) |  |
|  | yes | 11,041(69.23) | 1953(69.6) | 9,088(69.14) |  |
| Residence (N, %) |  |  |  |  | *<0.001* |
|  | rural | 10,608(66.5) | 2,088(74.5) | 8,520(64.8) |  |
|  | urban | 5,340(33.5) | 716(25.5) | 4,624(35.2) |  |
| Current drinking status (N, %) |  |  |  |  | *<0.001* |
|  | no | 10,012(62.8) | 1,655(59.0) | 8,357(63.6) |  |
|  | yes | 5,936(37.2) | 1,149(41.0) | 4,787(36.4) |  |
| Current smoking status (N, %) |  |  |  |  | *<0.001* |
|  | no | 10,443(65.5) | 1,671(59.6) | 8,772(66.7) |  |
|  | yes | 4,419(27.7) | 912(32.5) | 3,507(26.7) |  |
|  | missing | 1,086(6.8) | 221(7.9) | 865(6.6) |  |
| Depression (N, %) |  |  |  |  | *<0.001* |
|  | no | 10,824(67.9) | 1,675(59.7) | 9,149(69.6) |  |
|  | depressive symptoms | 4,269(26.8) | 869(31.0) | 3,400(25.9) |  |
|  | depression | 855(5.3) | 260(9.3) | 595(4.5) |  |
| Medical insurance (N, %) |  |  |  |  | *0.907* |
|  | no | 689(4.3) | 120(4.3) | 569(4.3) |  |
|  | yes | 1,5259(95.7) | 2,684(95.7) | 12,575(95.7) |  |
| Financial support from children (quintile, N, %) |  |  |  |  | *<0.001* |
|  | 1 | 5,326(33.4) | 749(26.7) | 4,577(34.8) |  |
|  | 2 | 2,949(18.5) | 609(21.7) | 2,340(17.8) |  |
|  | 3 | 3,423(21.5) | 669(23.9) | 2,754(21.0) |  |
|  | 4 | 3,864(24.2) | 691(24.6) | 3,173(24.1) |  |
|  | missing | 386(2.4) | 86(3.1) | 300(2.3) |  |
| Family size (Mean, SD) |  | 3.35(1.67) | 3.15(1.58) | 3.40(1.69) | *<0.001* |
| Potential caregivers (Mean, SD) |  | 0.07(0.26) | 0.26(0.44) | 0.03(0.18) | *<0.001* |

*Notes:* CHARLS, China Health and Retirement Longitudinal Study; SD, standard deviation; N, number; %, percentage; obs, observations.

**Table S 3.** Longitudinal association of caregiving and frailty among the whole sample (N=3,987, observations=15,948)

| **Frailty** | | | | | | | | |
| --- | --- | --- | --- | --- | --- | --- | --- | --- |
|  | Model1 | Model2 | Model3 | Model4 | Model5 | Model6 | Model7 | Model8 |
| Care status | | | | | | | | |
| Intercept | 0.127***  (0.001) | 0.132***  (0.001) | 0.132***  (0.001) | 0.132***  (0.001) | 0.122***  (0.003) | 0.130***  (0.003) | 0.129***  (0.004) | 0.122***  (0.004) |
| Non-caregivers as ref. |  |  |  |  |  |  |  |  |
| Caregiver | 0.010***  (0.001) | 0.011***  (0.001) | 0.007**  (0.002) | 0.012***  (0.003) | 0.012***  (0.003) | 0.013***  (0.003) | 0.011***  (0.003) | 0.009**  (0.003) |
| Time | 0.018***  (0.000) | 0.003*  (0.001) | 0.003*  (0.001) | 0.004***  (0.001) | 0.004**  (0.001) | 0.004**  (0.001) | 0.003**  (0.001) | 0.004**  (0.001) |
| Time*time |  | 0.005***  (0.000) | 0.005***  (0.000) | 0.005***  (0.000) | 0.004**  (0.000) | 0.004***  (0.000) | 0.004***  (0.000) | 0.004***  (0.000) |
| Non-caregivers as ref. |  |  |  |  |  |  |  |  |
| Caregiver *time |  |  | 0.002  (0.001) | -0.008*  (0.004) | -0.008*  (0.004) | -0.008*  (0.003) | -0.009*  (0.004) | -0.006+  (0.003) |
| Caregiver *time *time |  |  |  | 0.003**  (0.001) | 0.003**  (0.001) | 0.003**  (0.001) | 0.003**  (0.001) | 0.002*  (0.001) |
| Care intensity | | | | | | | | |
| Intercept | 0.127***  (0.001) | 0.132***  (0.001) | 0.132***  (0.001) | 0.131***  (0.001) | 0.122***  (0.003) | 0.130***  (0.003) | 0.129***  (0.004) | 0.122***  (0.004) |
| Non-caregivers as ref. |  |  |  |  |  |  |  |  |
| Lower intensity | 0.006***  (0.002) | 0.008***  (0.002) | 0.005+  (0.003) | 0.010**  (0.004) | 0.011**  (0.004) | 0.011**  (0.004) | 0.009*  (0.004) | 0.008*  (0.004) |
| Moderate intensity | 0.013***  (0.002) | 0.013***  (0.002) | 0.008*  (0.003) | 0.011**  (0.004) | 0.012**  (0.004) | 0.012**  (0.004) | 0.011**  (0.004) | 0.008*  (0.004) |
| Higher intensity | 0.024***  (0.003) | 0.023***  (0.003) | 0.026***  (0.006) | 0.026***  (0.007) | 0.027***  (0.006) | 0.027***  (0.006) | 0.026***  (0.006) | 0.017**  (0.006) |
| Time | 0.018***  (0.000) | 0.003**  (0.001) | 0.003**  (0.001) | 0.004**  (0.001) | 0.004**  (0.001) | 0.004**  (0.001) | 0.003**  (0.001) | 0.004**  (0.001) |
| Time*time |  | 0.005***  (0.000) | 0.005***  (0.000) | 0.005***  (0.000) | 0.004***  (0.000) | 0.004***  (0.000) | 0.004***  (0.000) | 0.004***  (0.000) |
| Non-caregivers as ref. |  |  |  |  |  |  |  |  |
| Care intensity*time |  |  |  |  |  |  |  |  |
| Lower intensity |  |  | 0.002  (0.001) | -0.009+  (0.005) | -0.009+  (0.005) | -0.008+  (0.005) | -0.010+  (0.005) | -0.008  (0.005) |
| Moderate intensity |  |  | 0.003*  (0.002) | -0.001  (0.005) | -0.006  (0.005) | -0.006  (0.005) | -0.007  (0.005) | -0.005  (0.005) |
| High intensity |  |  | -0.002  (0.002) | -0.003  (0.009) | -0.005  (0.009) | -0.005  (0.009) | -0.005  (0.009) | 0.001  (0.009) |
| Care intensity*time*time |  |  |  |  |  |  |  |  |
| Lower intensity |  |  |  | 0.003*  (0.002) | 0.003*  (0.002) | 0.003*  (0.002) | 0.003*  (0.002) | 0.003+  (0.001) |
| Moderate intensity |  |  |  | 0.003+  (0.002) | 0.003+  (0.002) | 0.003+  (0.002) | 0.003+  (0.002) | 0.003  (0.002) |
| Higher intensity |  |  |  | 0.000  (0.003) | 0.001  (0.003) | 0.001  (0.003) | 0.001  (0.003) | -0.000  (0.003) |

*Notes:* Model 1: caregiving status and time; Model 2: Model 1 + quadratic term for time; Model 3: Model 2 + interaction between caregiving status and time; Model 4: model 3 + interaction between caregiving status and the quadratic term for time; Model 5: model 4 + age, gender, education level, residence, and current working status; Model 6: model 5 + current drinking and smoking status; Model 7: Model 6 + medical insurance, financial support from children, number of potential caregivers, and family size; Model 8 (fully adjusted model): Model 7 + depression.

^+^*P*<0.10, ^*^*p*<0.05, ^**^*p*<0.01, ^***^*p*<0.001.

**Figure S 2.** Trajectory of frailty index by caregiving status among the whole sample

*Note:* adjusted by all covariates.

**Figure S 3.** Trajectory of frailty index by caregiving intensity among the whole sample

**** *Note:* adjusted by all covariates.

**Figure S 4.** Trajectory of frailty index by caregiving status among females

*Note:* adjusted by all covariates.

**Figure S 5.** Trajectory of frailty index by caregiving intensity among females

*Note:* adjusted by all covariates.

**Figure S 6.** Trajectory of frailty index by caregiving status among males

*Note:* adjusted by all covariates.

**Figure S 7.** Trajectory of frailty index by caregiving intensity among males

*Note****:*** adjusted by all covariates.

**Table S 4.** Proportion of missingness across four waves (N=4,143, excluding outcome missing)

| Variables |  | Missingness |  |  |
| --- | --- | --- | --- | --- |
|  | Wave 2011 | Wave 2013 | Wave 2015 | Wave 2018 |
|  | N (%) | N (%) | N (%) | N (%) |
| Current working status | 31(0.75) | 8(0.19) | 5(0.12) | / |
| Current drinking status | / | 5(0.12) | 2(0.05) | / |
| Current smoking status | / | 1,127(27.20) | 2(0.05) | / |
| Medical insurance | 7(0.17) | 21(0.51) | / | / |
| Depression | 23(0.56) | 21(0.51) | 7(0.17) | 31(0.75) |
| Financial support from children | 26(0.63) | 365(8.81) | 7(0.17) | 7(0.17) |
